# First genome edited poinsettias: targeted mutagenesis of flavonoid 3′-hydroxylase using CRISPR/Cas9 results in a colour shift

**DOI:** 10.1007/s11240-021-02103-5

**Published:** 2021-05-26

**Authors:** Daria Nitarska, Robert Boehm, Thomas Debener, Rares Calin Lucaciu, Heidi Halbwirth

**Affiliations:** 1 Institute of Chemical, Environmental and Bioscience Engineering, Technische Universität Wien, 1060 Vienna, Austria; 2 Klemm+Sohn GmbH & Co, 70379 Stuttgart, Germany; 3 Institute of Plant Genetics, Leibniz Universität Hannover, 30419 Hannover, Germany

**Keywords:** *Euphorbia pulcherrima*, Orange bract colour, Pelargonidin, Breeding

## Abstract

The CRISPR/Cas9 system is a remarkably promising tool for targeted gene mutagenesis, and becoming ever more popular for modification of ornamental plants. In this study we performed the knockout of *flavonoid 3*′*-hydroxylase* (*F3*′*H*) with application of CRISPR/Cas9 in the red flowering poinsettia (*Euphorbia pulcherrima)* cultivar ‘Christmas Eve’, in order to obtain plants with orange bract colour, which accumulate prevalently pelargonidin. F3′H is an enzyme that is necessary for formation of cyanidin type anthocyanins, which are responsible for the red colour of poinsettia bracts. Even though F3′H was not completely inactivated, the bract colour of transgenic plants changed from vivid red (RHS 45B) to vivid reddish orange (RHS 33A), and cyanidin levels decreased significantly compared with the wild type. In the genetically modified plants, an increased ratio of pelargonidin to cyanidin was observed. By cloning and expression of mutated proteins, the lack of F3′H activity was confirmed. This confirms that a loss of function mutation in the poinsettia *F3′H* gene is sufficient for obtaining poinsettia with orange bract colour. This is the first report of successful use of CRISPR/Cas9 for genome editing in poinsettia.

**Supplementary Information:**

The online version contains supplementary material available at 10.1007/s11240-021-02103-5.

## Key message

Genome edited poinsettia plants show loss-of-function mutations in the F3’H gene introduced by CRISPR/Cas9. This is sufficient to change the bract colour from red to reddish orange.

**Supplementary Information:**

The online version contains supplementary material available at 10.1007/s11240-021-02103-5.

## Introduction

The winter-flowering *Euphorbia pulcherrima* (poinsettia or Christmas Star) belongs to the most economically important potted ornamental plants, especially during the Christmas season (Ecke 2004). Traditionally, the mass market prefers intense scarlet or dark-red colouration of the bracts (Taylor et al. 2011), the latter being leaves that change their colour from green to red to support the plain cymes in pollinator attraction. However, during the last two decades, a huge diversity of red hues and novelty colours and styles has arisen in the poinsettia assortment, because a considerable number of consumers is willing to pay higher prices for unusual varieties (Barrett 2005). Among these, orange-red bract colour seems to attract European and North-American consumers, and has the potential to extend the market e.g. as a Halloween speciality (Barrett 2005).

**Fig. 1:**
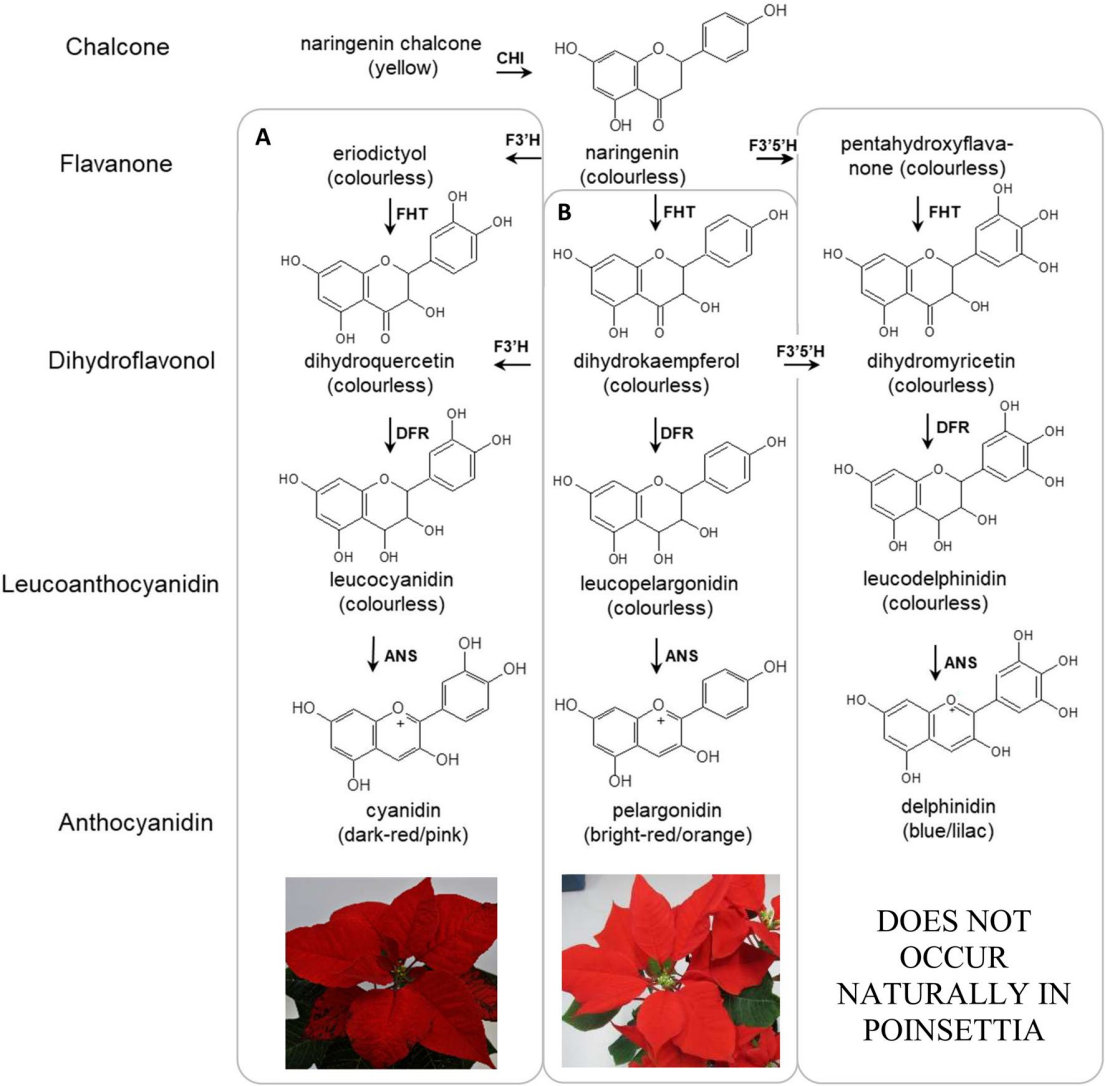
Simplified overview of the anthocyanin pathway. Abbrev: ANS: anthocyanidin synthase, CHI: chalcone isomerase, DFR: dihydroflavonol 4-reductase, FHT: flavanone 3-hydroxylase, F3′H: flavonoid 3′-hydroxylase, F3′5′H: flavonoid 3′,5′-hydroxylase. **a**: The prevalent pathway in naturally occurring poinsettia; **b**: The chosen strategy for attaining reddish orange bracts

The pigments responsible for poinsettia bract colouration, at least for the red hues, are the anthocyanins, a well-known group of secondary metabolites (Stewart et al. 1979; Slatnar et al. 2013). Two main types of anthocyanins can be distinguished in poinsettia, based on the number of hydroxyl groups in the B-ring, the pelargonidin type (one hydroxyl group) and the cyanidin type (two hydroxyl groups). Recently, we have shown that the rare orange-red bract colouration of poinsettia (*Euphorbia pulcherrima*) is associated with a prevalence of pelargonidin-type anthocyanins and a somewhat decreased anthocyanin concentration in general. We also reported that accumulation of pelargonidin-based pigments in poinsettia is caused by different mechanisms, of which one is a strong reduction of flavonoid 3′-hydroxylase (F3′H) activity in the bracts (Nitarska et al. 2018). F3′H (EC 1.14.13.21) is a membrane bound enzyme associated with the cytosolic site of the endoplasmic reticulum (Schuler and Werck-Reichhart 2003) and belongs to the subfamily CYP75B of cytochrome P450-dependent monooxygenase (P450) (Tanaka 2006; Chapple 1998). F3′H is responsible for the introduction of a second hydroxyl group in the B-ring thereby leading to the formation of cyanidin type anthocyanins (Fig. 1a). F3′H, however, does not act on anthocyanins themselves, but on one of the intermediates upstream of the anthocyanin forming step (Schwinn et al. 2014). Particularly, the number of hydroxyl groups in the B-ring of the dihydroflavonol precursors determines the colour of each group of anthocyanins. Dihydrokaempferol (DHK), with one hydroxyl group, is converted to orange-red pelargonidin type anthocyanins, and dihydroquercetin (DHQ), with two hydroxyl groups, to red-pink cyanidin type (Halbwirth 2010) (Fig. 1). As has been described (Nakatsuka et al. 2007), silencing of *F3’H* is an important factor for obtaining plants that accumulate prevalently pelargonidin type anthocyanins in tobacco.

Recently, the Clustered Regularly-Interspaced Short Palindromic Repeats (CRISPR/Cas9) system was reported as a promising tool for targeted genome modification in plants (Corte et al. 2019) and it has quickly developed into the most widely used genome editing method (Kishi-Kaboshi et al. 2018). In this system, double-strand breaks (DSBs) are introduced by Cas9 nuclease, guided by a 20 nucleotide sequence, single guide RNA (sgRNA) (Jinek et al. 2012). There are two mechanisms of DSB repair, homology recombination (HDR) and non -homologous end joining (NHEJ) (Puchta 2005). The second mechanism usually leads to the introduction of a mutation, insertion or deletion, in the gene sequence and consequently to a loss of function in the protein (Chiruvella et al. 2013). This approach is now commonly used for specific gene knockout in many plant species (Ma et al. 2016). For the first time, the CRISPR/Cas9 system was used in ornamentals for silencing *phytoene desaturase* (*PDS)* in petunia (*Petunia x hybrida)* (Zhang et al. 2016). The first successful alteration of flower traits was reported in Japanese morning glory (*Ipomoea nil*), where *dihydroflavonol 4–reductase* (*DFR*) was targeted (Watanabe et al. 2017). Currently this approach is becoming more and more popular for the modification of ornamentals. Up to now it was used for targeting multiple secondary metabolism genes in orchid (*Phalaenopsis*) (Tong et al. 2020), chrysanthemum (*Chrysanthemum morifolium)* (Kishi-Kaboshi et al. 2017), lily (*Lilium pumilum*, *Lilium longiflorum*) (Yan et al. 2019), torenia (*Torenia fournieri*) (Nishihara et al. 2018) and gentian (*Gentiana triflora* × *Gentiana scabra*) (Tasaki et al. 2019).

This study is a proof of concept for the conclusion from our previous work (Nitarska et al. 2018), that a mutation in the *F3*′*H* gene is sufficient to obtain pelargonidin accumulating poinsettia. We attempted to knockout the poinsettia *F3*′*H* gene by applying the CRISPR/Cas9 method to obtain poinsettias prevalently accumulating pelargonidin type anthocyanins in their bracts (Fig. 1b). To our knowledge, this is the first report of a genome-edited poinsettia.

## Materials and methods

### Chemicals

(2-^14^C)-Malonyl-coenzyme A (55 mCi mmol^−1^) was purchased from New England Nuclear Corp. GmbH (Vienna, Austria). Synthesis of radiolabeled substrates was performed as described in (Halbwirth et al. 2006). Reference substances for HPLC analysis (cyanidin, pelargonidin, delphinidin, malvidin, petunidin,) were purchased from Extrasynthese (Genay, France).

### Plant material

Poinsettia *Euphorbia pulcherrima* cultivar ‘Christmas Eve’ (Klemm + Sohn GmbH & Co. KG, Germany) was used for transformation. Plants were grown in the greenhouse under long day conditions (16 h day/8 h night). For *Agrobacterium*-mediated transformation internode stem explants were used. Excised stems were surface sterilized by washing for 10 min in 1.5% solutions of NaOCl with 1 drop of Tween 20 and then washed twice for 10 min in sterile water. In the next step, internode stems were cut (around 1 mm thick) and placed on callus induction media [CIM–MS media supplemented with 0.2 mg L^−1^ 4-chlorophenoxy acetic acid (CPA) and 0.2 mg L^−1^ 6-Benzylaminopurine (BAP)] for 4–5 days and then explant discs were used for transformation.

### sgRNA design and cloning

**Fig. 2:**
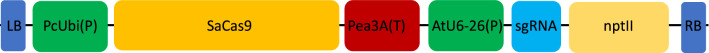
Schematic diagram of the pDe-Sa_Cas9 expression cassette. *PcUbi* promoter, *SaCas9 Staphyloccocus aureus* Cas9, *Pea3A Pisum sativum* Pea3A terminator, *AtU6-26 Arabidopsis thalaina* U6-26 promoter, *sgRNA* single guide RNA scafold, *nptII* kanamycin resistance cassette

For transformation, the binary vector pDe-Sa_Cas9 (Steinert et al. 2015), carrying a kanamycin resistance gene (*nptII*) to facilitate selection was used (Fig. 2). A sgRNA sequence was designed based on the sequences available in the Gene Bank (KY273440.1), with application of the online tool CHOPCHOP (Labun et al. 2016; Montague et al. 2014). A 20 nucleotide long sgRNA sequence (CAGTCAATAGCCTCCTTGGC), without PAM sequence (TCGGGT), was cloned into a pEN-Sa_Chimera vector (primers for cloning: pEN-Sa_Chim_EpF3HgRNA_F ATTGCAGTCAATAGCCTCCTTGGC and pEN-Sa_Chim_EpF3HgRNA_R AAACGCCAAGGAGGCTATTGACTG) (Steinert et al. 2015). In the second step, a sgRNA expression cassette was transferred to the destination vector (pDe-Sa_Cas9) by single-site Gateway LR reaction (Thermo Fisher Scientific https://www.thermofisher.com/pl/en/home/life-science/cloning/gateway-cloning/protocols.html). Cloning success was confirmed by sequencing by a commercial supplier (LGC, Germany).

### Transformation and regeneration

*Agrobacterium tumefaciens* strain GV3101, carrying the pDe-Sa_Cas9 vector with cloned sgRNA was cultivated in SOB media (2% w/v tryptone, 0.5% w/v yeast extract, 10 mM NaCl, 2.5 mM KCl, 10 mM MgCl_2_, 10 mM MgSO_4_) supplemented with 50 mg L^−1^ rifampicin, 100 mg L^−1^ spectinomycin and 30 mg L^−1^ gentamicin for 24 h with shaking (200 rpm) at 28 °C. Then 5 mL of this culture was used to inoculate 50 ml of Minimal A medium (10.5 g L^−1^ K_2_HPO_4_, 4.5 g L^−1^ KH_2_PO_4_, 1 g L^−1^ (NH_4_)_2_SO_4_, 0.52 g L^−1^ Na_3_C_6_H_5_O_7_ × 2 H_2_O, 0% w v^−1^ glucose, 0.005% MgSO_4_ × 7 H_2_0, 0.00025% thiamine) and was further cultivated at identical conditions till OD reached 0.5. Then bacteria were used for transformation. Explants were incubated with 10 mL of *Agrobacterium* inoculum for 30 min with gentle shaking and dried on sterile paper, then placed on the CIM for 2 days of co-cultivation. As control explants were incubated with 10 mL of Minimal A medium. Subsequently, discs were washed in sterile water containing the antibiotics 250 mg L^−1^ cefotaxim and 150 mg L^−1^ timentin for 30 min and dried on sterile paper, then placed on the CIM supplemented with 250 mg L^−1^ cefotaxim and 150 mg L^−1^ timentin for callus induction. After 14 days on CIM, explants were transferred to somatic embryo induction media [SEIM–MS medium with 0.2 mg L^−1^ 1-Naphthaleneacetic acid (NAA) and 0.1 mg L^−1^ isopentenyl adenine (2ip)] supplemented with 250 mg L^−1^ cefotaxim, 150 mg L^−1^ timentin and 2.5 mg L^−1^ kanamycin, for induction of somatic embryogenesis. After 3–6 weeks, when somatic embryos were visible, explants were transferred to somatic embryo maturation medium (SEMM–MS medium with 0.05 mg L^−1^ BAP) supplemented with 250 mg L^−1^ cefotaxim, 150 mg L^−1^ timentin and 200 or 50 mg L^−1^ kanamycin for further cultivation and selection. Fully regenerated plants were placed on MS media without antibiotics, propagated and transferred to the greenhouse. Plants were transferred from the medium into paper-wrapped substrate plugs and carefully adapted to greenhouse conditions. After hardening, plugs were potted in 1 L-pots containing Einheitserde P substrate (Hermann Meyer KG, Germany) and grown with an average temperature of 22 °C under natural light conditions. Three cuttings per line were grafted onto Phytoplasma-containing rootstock to promote branching of the scion. One successfully grafted scion per line was further developed as mother plant to generate cuttings for vegetative propagation. At least 5 cuttings per line were rooted and potted into 1 L-pots containing Einheitserde P substrate (Hermann Meyer KG, Germany). They were cultivated with an average temperature of 22 °C under short-day conditions (9 h of daylight, 15 h of darkness) to induce flower formation and to stimulate the development of colored bracts.

### Screening of regenerated plants

Genomic DNA was extracted from leaves of regenerated poinsettia with InVisorb Plant mini kit (Stratec, Germany). Transgenic plants were selected by PCR, where presence of *nptII*, *cas9* and sgRNA sequences was detected (primers’ sequences are provided in Suppl. Table S1). For screening, Go*Taq* polymerase (Promega, Germany) was used. The reaction contained a final volume of 20 µL: 4 µL 5 × Go*Taq* Green buffer, 0.4 µL dNTPs, 1 µL forward primer (10 µM), 1 µL reverse primer (10 µM), 2 µL DNA, 0.2 µL *Taq*; reaction conditions: 2 min 94 °C initial denaturation, 40 cycles (98 °C, 30 s.denaturation, 60/62 °C, 30 s. primer annealing, 72 °C, 45 s. extension), 10 min 72 °C final extension. From positive plants, the total RNA was extracted by mirPremier Kit (Sigma Aldrich, Austria) according to the protocol provided. cDNA was synthesized with RevertAid H Minus Reverse Transcriptase (Thermo Scientific, US) according to the manufacturer’s protocol. Primers and PCR conditions were the same as described above. Integrity of DNA as cDNA was confirmed by amplification of poinsettia endogenous genes (*actin*, *GAPDH* or Ep*F3*′*H* fragment).

### Sequence analysis

Genomic DNA from genome-edited plants’ leaves was extracted using DNeasy plant mini kit (Qiagen, Germany) according to the manufacturer’s protocol. Gene fragments were amplified with EpF3′HpYes-F and EpF3′H-crispr-R primers (Suppl. Table S1) with Q5 High Fidelity DNA Polymerase (New England Biolabs, Austria). PCR program: 30 s. 98 °C for initial denaturation, 30 cycles (98 °C 30 s. denaturation, 62 °C 30 s. primer annealing, 72 °C 30 s. extension), 2 min 72 °C final extension. PCR products were separated on 1% agarose and extracted using Wizard SV Gel and PCR Clean-up System (Promega, Germany). Sub-libraries were constructed with application of NEBNext Ultra II DNA Library Prep kit for Illumina (New England Biolabs, Austria) according to the manufacturer’s protocol. For indexing, NEBNext Multiplex Oligos for Illumina (Index Primer Set 1) (New England Biolabs, Austria) were used. Concentration of each sub-library was measured with Qubit (Invitrogen, USA). The bulked library was obtained by mixing equal amounts of each sub-library. The concentration of the bulked library was measured with Qubit and checked on Fragment Analyzer (Agilent, USA), and then it was diluted to 8 pM. As a control PhiX (Illumina, USA) was spiked into bulked library in a concentration of 1.3%. Sequencing runs were performed on Illumina MiSeq system, with application of MiSeq v2 Reagent Kit 300 cycles (Illumina, USA) (2 × 150 reads). Sequencing results were analysed with CRISPResso2 software (Clement et al. 2019).

### HPLC analysis

Poinsettia bract pigment analysis was performed by HPLC as previously described (Haselmair-Gosch et al. 2018). For the extraction, 0.5 g of shock frozen poinsettia bracts were used in 1.5 mL of 2 M HCl in methanol.

### Gene expression studies

The *F3′H* and *Cas9* gene expression was evaluated by qPCR using the StepOnePlus system (Applied Biosystems, Germany) and the Luna® Universal qPCR Master Mix (New England Biolabs, Austria) according to the manufacturers’ protocols. The analysis was performed in three independent replicates and the results were normalized to the two control genes, *actin* and *translation elongation factor I alpha* (*EF1 A*) (Zhang et al. 2013). Primer efficiency (Suppl. Table S1) and the relative expression ratio were calculated according to Pfaffl (Pfaffl 2004). Product specificity was confirmed by analysis of melting curves.

### Poinsettia F3’H cloning and heterologous expression in yeast

F3′H gene from transgenic plants and WT were cloned into pYes2.1/V5-His-TOPO vector (Invitrogen, US) using EpF3′HpYes-F and EpF3′HpYes-R primers (Suppl. Table S1). Heterologous expression in yeast and the F3′H enzyme assay was performed as previously described (Nitarska et al. 2018).

### Statistical analysis

The statistical analysis was performed in GraphPad Prims version 8.4.3 for Mac. Normality of data was checked with the Shapiro–Wilk test. Statistical significance was calculated using a double-tailed, unpaired *t*-test when the variances were equal or unpaired *t*-test with Welch’s correction when variances were different. The Mann–Whitney test was used for not normally distributed data.

## Results

### Transformation and regeneration

In order to obtain poinsettia plants with inactive *F3*′*H*, a CRISPR/Cas9 approach was used. Poinsettia stem segments were transformed via *Agrobacterium*-mediated transformation with the pDe-Sa_Cas9 vector carrying a sgRNA sequence specific to poinsettia *F3*′*H* (Fig. 2). For transformation, cultivar ‘Christmas Eve’ was selected as the best for regeneration among four other tested cultivars (data not shown). In total, seven experiments were performed, which resulted in the regeneration of 105 putatively transgenic plants (Table 1). Due to the fact that the stem segments originated from the greenhouse and the surface sterilization process was not always sufficient, a huge number of explants was lost during the first days after transformation, due to fungal and bacterial infections. Although the callus formation process was very efficient, the embryo formation could be observed only on a few explants. In experiments A to D, 200 mg L^−1^ of kanamycin was used for selection, which resulted in the regeneration of only 7 plants, because most of the embryos and plantlets did not survive the selection step. In order to obtain more regenerated plants in experiments E to G we lowered the kanamycin content to 50 mg L^−1^ as was suggested in literature (Clarke et al. 2008). At these conditions, we obtained an additional 98 putatively transformed plants, which would suggest that the previous selection conditions could have been too harsh for the cultivar ‘Christmas Eve’.

**Table 1:** Overview of the transformation of the poinsettia cultivar ‘Christmas Eve’

Transformation event	Kanamycin concentration [mg L^−1^]	Explants	Lost explants	Embryogenic explants	Regenerated plants
A	200	46	7	36	3
B	200	349	235	49	4
C	200	66	66	0	0
D	200	446	337	0	0
E	50	389	335	18	20
F	50	275	241	31	25
G	50	504	216	113	53
Total	–	1975	1437	247	105

### Screening of regenerated plants

**Fig. 3:**
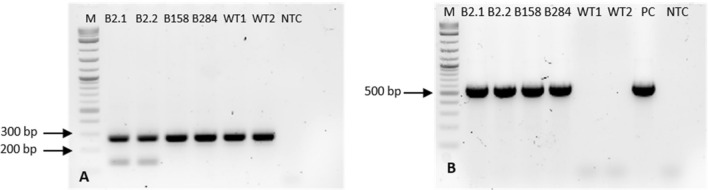
PCR evaluation of the transgenic plants and WT control on gDNA level. **a**: Amplification of Ep*F3’H* fragment (primers EpF3’HpYesF and EpF3’HcrisprR), **b**: Amplification of *cas9* fragment (primers Cas9-F1 and Cas9-R1). *M* molecular size standard 1 kb Plus DNA ladder (New England Biolabs, Austria), *PC* positive control (plasmid), *NTC* non template control. Selected fragments of ladder are labelled for better orientation

**Fig. 4:**
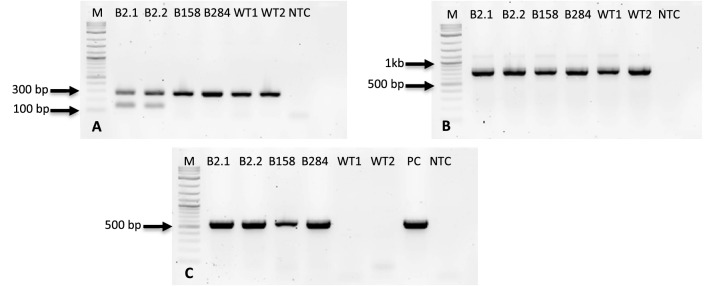
PCR evaluation of the transgenic plants and WT on cDNA level. **a**: Amplification of Ep*F3’H* fragment (primers EpF3’HpYesF and EpF3’HcrisprR), **b**: Amplification of Ep*GAPDH* fragment (primers EpGAPD-F and EpGAPDH-R), **c**: *cas9* fragment (primers Cas9-F1 and Cas9-R1). *M* molecular size standard 1 kb Plus DNA ladder (New England Biolabs, Austria), *PC* positive control (plasmid), *NTC* non template control. Selected fragments of ladder are labelled for better orientation

Screening of regenerated plants was performed in two steps. In the first step, genomic DNA from leaves of regenerated plants was extracted and the presence of the transgene was investigated by PCR (targets: *nptII*, *cas9*). In step II, the total RNA was extracted from plants harbouring the transgene as identified in step I. Thereafter, cDNA was synthesized and used as a template for PCR (targets: *nptII*, *cas9*, gRNA) to check the expression of the transgene. In the first screening step, a total of 14 positive plants were found (Table 2, Fig. 3). The high number of negative plants (91) can be attributed to the decrease of the kanamycin concentration in the SEM media, which caused a bigger number of escapes. The second step of screening revealed that expression of the transgene could be detected only in three plants, which all originated from experiment B (200 mg L^−1^ kanamycin) (Fig. 4). Positive lines named B2, B158 and B284 were transferred to the greenhouse for further cultivation.

**Table 2:** Regenerants screening summary

Transformation event	Total number of plants	Positive DNA	Positive RNA	Dead
*A*	3	0	0	0
*B*	4	4	3	1
*C*	0	0	0	0
*D*	0	0	0	0
*E*	20	4	0	2
*F*	25	1	0	1
*G*	53	5	0	1
*Total*	105	14	3	5

### Sequence analysis

To analyse the *F3*′*H* sequence of transgenic plants, next generation sequencing (NGS) was performed. 24% of the reads for line B2 reddish orange (B2.1) and 19% of line B2 chimera (B2.2) was modified (Table 3). For lines B158 and B284, almost no modifications were found. The most prevalent modification for line B2 was an insertion of a thymine (T) located three nucleotides before the PAM sequence. Nevertheless, in all transgenic lines, the most prevalent sequence was that of the WT, which suggests that the genome editing approach was not efficient enough. Even for line B2 more that 66% reads were identical to the sequence of the WT, which explains the presence of cyanidin type anthocyanins in the bracts. Interestingly, only a slightly higher number of WT reads was detected for line B2 chimeric plants in comparison to the fully reddish orange B2 plant. In all analysed plants, also other sequences than the major reads were present and typically consisted of 0.5% of total reads or less. This outcome is considered to be a result of PCR and NGS errors. Sequencing analysis confirmed that the genome editing in line B2 worked, but still the WT sequence is present, which results in just a partial shift toward pelargonidin in anthocyanin synthesis for this transgenic line.

**Table 3:** NGS analysis of poinsettia *F3*′*H* amplicons of genome-edited lines

Line	Total number of reads	Number of reads	%	Targeted F3′H sequence	In/Del
WT	2,327,800	2,185,214	93.87	CAGTCAATAGCCTCCTTGGC**TCGGGT**	
B2.1	2,326,081	1,551,158 552,471	66.69 23.75	CAGTCAATAGCCTCCTTGGC**TCGGGT** CAGTCAATAGCCTCCTT*T*GGC**TCGGGT**	WT + 1
B2.2	1,646,282	1,176,195 316,765	71.45 19.24	CAGTCAATAGCCTCCTTGGC**TCGGGT** CAGTCAATAGCCTCCTT*T*GGC**TCGGGT**	WT + 1
B158	3,096,423	2,935,697	94.81	CAGTCAATAGCCTCCTTGGC**TCGGGT**	WT
B284	3,097,207	2,925,612	94.46	CAGTCAATAGCCTCCTTGGC**TCGGGT**	WT

Inserted nucleotides are marked in italics, PAM sequence (TCGGGT) is marked in bold

### Bract analysis

**Fig. 5:**
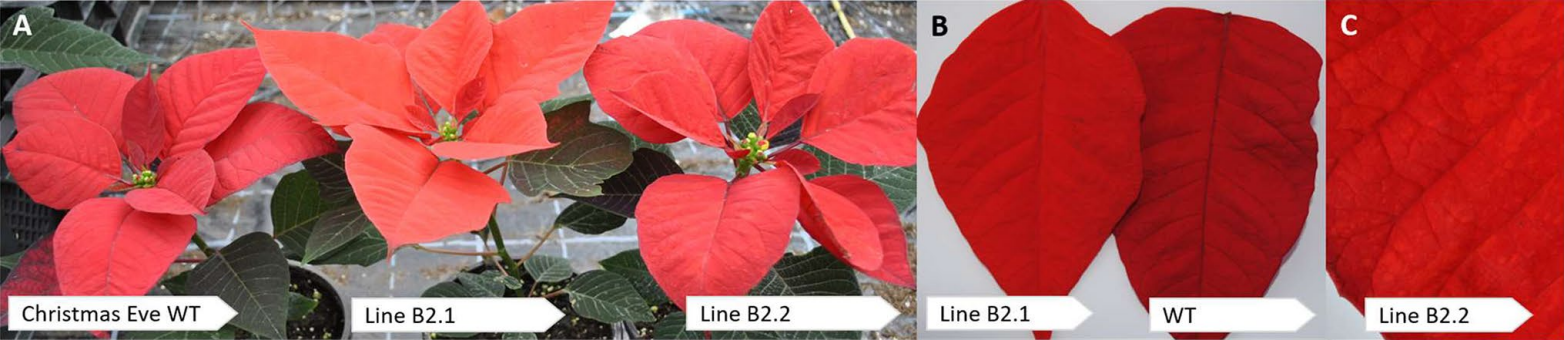
Bract colours of WT and genome-edited poinsettias. **a**: ‘Christmas Eve’ WT (left); B2.1 (centre), B2.2 (right); **b**: B2.1 (left), Christmas Eve WT (right); **c**: B2.2, Close-up showing chimeric phenotype on bracts (reddish-orange patches on dark red background)

Bract colouration was induced by cultivation for 8 weeks at short day conditions (11 h day, 13 h night). In the case of line B2, two plants were obtained by propagation of the regenerated shoot, of which one showed bracts with brighter colour (hereafter named B2.1) compared with the wild type (WT) (Fig. 5a, b). The second plant (hereafter named B2.2) showed scattered points of brighter colour on the bracts, which could be described as chimeric phenotype (Fig. 5c). For lines B158 and B284, one plant each was obtained, which showed the same phenotype as the WT. All lines after first blooming were further propagated and analysed. The colour of propagated plants of line B2.1 was assigned to groups between 33A and 44B on the RHS charts (5th edition 2007), both are described as vivid reddish orange, but 33A is placed in the orange colour group and 44B in the red colour group. Wild type ‘Christmas Eve’ was assigned to group 45B, described as vivid red.

**Fig. 6:**
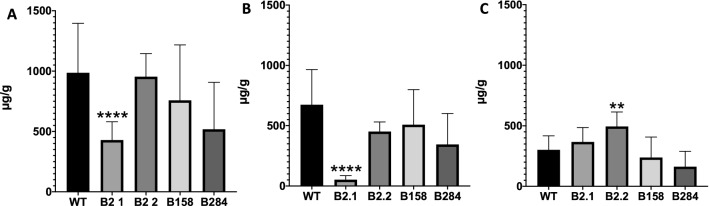
Anthocyanins in bracts of poinsettia. **a**: Total anthocyanin content in transgenic lines and WT control, **b**: Cyanidin content, **c**: Pelargonidin content. Data were calculated from at least four biological repetitions and with error bars representing standard deviation. Statistical significance ^*^p < 0.05, ^**^p < 0.01, ^***^p < 0.001, ^****^p < 0.0001

To analyse the anthocyanin content in the transgenic poinsettia bracts, HPLC analysis was performed. The total amount of anthocyanins in bracts was lower for line B2.1 with fully reddish orange bracts (Fig. 6a). The fully reddish orange plants of line B2.1 had a significantly lower amount of cyanidin compared with the WT (Fig. 6b). In all other lines, the cyanidin level was only slightly lower than in the WT (Fig. 6b). The pelargonidin amount, however, in almost all transgenic lines was not significantly different. Only for line B2.2 the increase of the pelargonidin content was observed (Fig. 6c).

### Gene expression studies

**Fig. 7:**
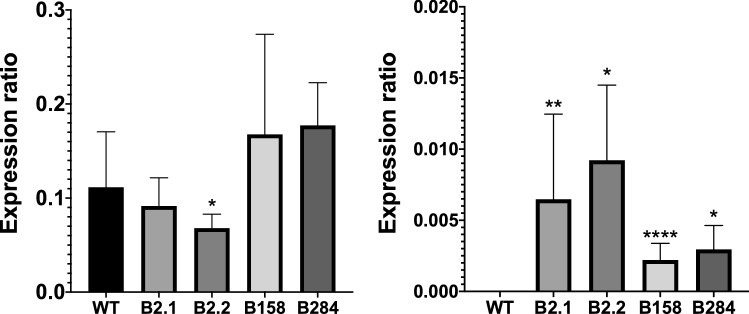
Gene expression of poinsettia *F3’H* (left) and *Cas9* (right) in transgenic poinsettia and WT. Quantitative expression was normalized to *actin*. Data were calculated from at least four biological repetitions and with error bars representing standard deviation. Statistical significance ^*^p < 0.05, ^**^p < 0.01, ^***^p < 0.001, ^****^p < 0.0001

The expression level of poinsettia *F3*′*H* and the *Cas9* gene, in the transgenic plants and the WT ‘Christmas Eve’ was determined by quantitative real-time PCR using *actin* and *EF1A* for normalization (primer sequences Suppl. Table S1). In general, the expression level of poinsettia *F3*′*H* for all transgenic lines was on a comparable level as in the WT, only for line B2.2 was it slightly lower (Fig. 7). We were able to detect *Cas9* expression in all transgenic lines, whereas in the WT it was not observed. In all lines, expression of *Cas9* between the propagated plants varied widely. The highest values were measured for line B2, whereas for the two other lines it was two or four times lower (Fig. 7). This correlates with the fact that a changed colouration could be observed only in line B2. In the other two lines (B158 and B284), the expression of *Cas9* was probably not sufficient to perform the edit of the targeted gene.

### F3’H cloning and heterologous expression in yeast

**Fig. 8:**
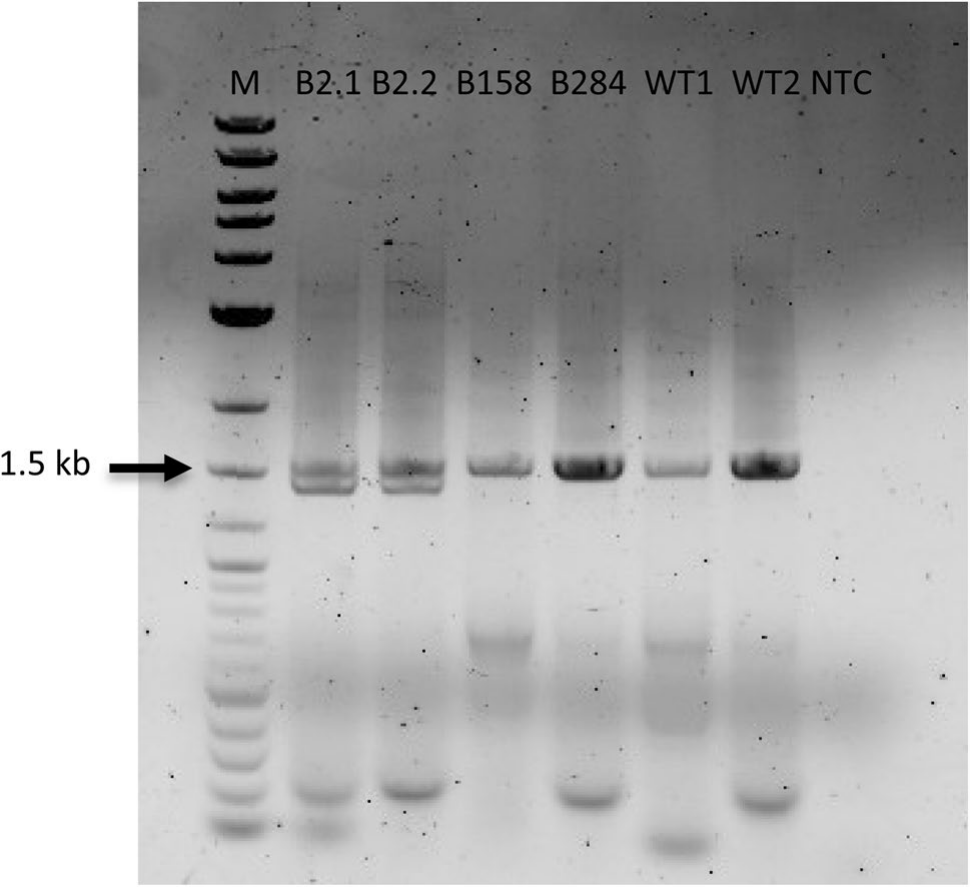
PCR amplification of full-size poinsettia *F3’H* with primers EpF3’HpYesF and EpF3’HpYesR and cDNA as a template*. M* molecular size standard 1 kb Plus DNA ladder (New England Biolabs, Austria), *NTC* non template control. 1.5 kb fragment of the size marker is labelled for better orientation

After PCR amplification of full size *F3’H* from transgenic plants from lines B2.1 and B2.2, two bands were detected (Fig. 8). One band with the original size present in the wild type around 1.5 kb, and second which was approx. 100 base pairs smaller. The same result was observed during screening, when also two bands were obtained when *F3*′*H* fragment was amplified as endogenous control (Fig. 3a, Fig. 4a). Therefore, *F3*′*Hs* from all transgenic plants and the WT were isolated and heterologously expressed in yeast in order to check the recombinant proteins for functional activity.

**Fig. 9:**
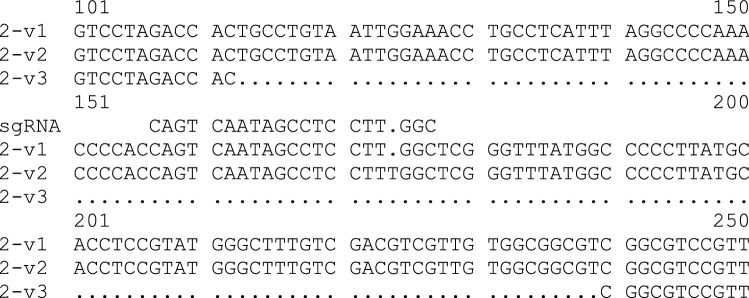
Multiple nucleotide alignment of three *F3’H* versions from line B2 and the sgRNA sequence

From the line B2, three different versions of F3′H cDNA clones were isolated, version 1 (v1) with a sequence identical to that of the WT, version 2 (v2) showing an insertion of a T in position 171, 3 nucleotides upstream PAM sequence, and version 3 (v3) showing a deletion of 126 nucleotides (Fig. 9, Suppl. Fig S1). *F3*′*Hs* clones isolated from lines B158 and B284 were identical to those of the WT (Suppl. Fig. S1, S2). In *F3*′*H* v2, the insertion of an additional T caused a shift in the open reading frame and thus, a premature termination of protein synthesis, whereas the deletion in *F3*′*H* v3, results in the loss of 42 amino acids between positions 37 and 79 (Suppl. Fig. S2). However, recombinant F3′Hs produced from *F3*′*H* v2 and v3 were not functionally active, which was tested with two main substrates of F3′H. All other recombinant F3′Hs were active, and the conversion of the substrate was on a similar level as that of the F3′Hs present in the WT (Table 4).

**Table 4:** Functional activity test of recombinant F3′Hs from transgenic plants

Name	Naringenin conversion (%)	DHK conversion (%)
WT	96	82
B2-v1	83	78
B2-v2	0	0
B2-v3	4	0
B158	84	81
B284	89	42

## Discussion

Over more than 200 years of cultivation, the appearance of poinsettia has changed tremendously. Wild poinsettias are shrubs or small trees of up to 3 m height, with long internodes, a few stems, and narrow leaves and bracts (Trejo et al. 2012; Lee 2000). Commercial poinsettias as we know them from flower shops and supermarkets, in contrast, are small compact potted plants with multiple branches and wide large bracts (Trejo et al. 2012). The development of modern poinsettia took place in the twentieth century and the main milestones were the introduction of photoperiodic control, branching induction by transfer of phytoplasma via grafting, and the use of growth regulators (Taylor et al. 2011).

The colour spectrum of the bracts has also changed over time. Wild poinsettias are red or in a few cases white, but nowadays poinsettia are available in many different colour variations (Taylor et al. 2011), which gain more and more popularity (Barrett 2005). Apart from crossbreeding, radiation mutation is one of the basic methods to obtain novel bract colours (Broertjes and Harten 2013). The main drawback of this method is that mutations occur randomly, and a large number of plants has to be screened for interesting novel phenotypes (Kishi-Kaboshi et al. 2018), although in poinsettia the mutation rates leading to changes in the flower colour is relatively high (Lee et al. 2010). Genome editing methods like the CRISPR/Cas9 system offer significant improvement, as these allow precise targeting of the gene to be mutated (Liu et al. 2017). An additional advantage of CRISPR/Cas9 is that the transgene can be segregated in the progeny, so that a mutated plant without presence of a transgene can be obtained (Gao et al. 2016). CRISPR/Cas9 constructs are usually transferred to the plant cells by *Agrobacterium*- mediated transformation or particle bombardment (Erpen-Dalla Corte et al. 2019), which is a challenging process in some plant species.

Up to now, three classical poinsettia transformations mediated by *Agrobacterium* (Clarke et al. 2008) (Islam et al. 2013) (Sagvaag 2015) have been reported. To our knowledge, our approach is the first attempt to combine *Agrobacterium*- mediated transformations with CRISPR/Cas9 application in poinsettias. The approach faced problems with explant contamination due to insufficient surface sterilization. Clarke et al. (2008) suggested that the latex sap occurring at poinsettia cutting spots makes surface sterilization more challenging. The regeneration process of the chosen cultivar Christmas Eve was satisfactory. The percentage of somatic embryo producing explants was, however, lower than reported by Clarke et al. (2008) for cv. Millennium and is probably strongly dependent on the cultivar. The total transformation frequency during the transformation of cv. Millennium (Clarke et al., 2008) was similar to the value obtained in this study and it is around 2% (considering only explants that were not lost due to contamination).

Despite the fact that the transgene was detected in 14 regenerated plants on the genomic DNA level, only 3 of them showed transgene expression (6 were lost during the cultivation process). Of these, just one line (B2) developed bracts with brighter, reddish orange colour, than the WT (Fig. 5). When plants were propagated from the original shoots of B2, chimeric types were obtained in addition to the fully bright reddish orange phenotype. The chimeric plants can be described as non-pattern sectorial chimeras. This kind of mosaic phenotype is unstable (Frank and Chitwood 2016) and during further propagation, plants with different amounts of brighter areas were obtained. The occurrence of chimeric phenotypes was also reported for Japanese morning glory, in which *DFR* had been knocked out with the application of CRISPR/Cas9 system (Watanabe et al. 2017), and torenia where *F3H* was silenced (Nishihara et al. 2018). Probably chimeric tissue is a result of ongoing genome editing in line B2, despite that most of the genome edition events is considered to take place during early transformation stage (Nishihara et al. 2018). Only possibility to stop this process is crossing out *Cas9*, what is a problem in vegetatively propagated plants like poinsettia (Taylor et al. 2011). Chimerism of line B2 was confirmed by NGS data, when WT reads were detected together with mutated sequence (+ 1 nucleotide) in both fully reddish orange and the chimeric plant and by cloning of three versions of F3’H which were expressed in line B2. Lines B158 and B284 produced bracts with the same colour as the WT, indicating that the transgene could have been silenced. NGS data showed that only WT type reads are present in both lines indicating that the edition did not occur in those lines. This can happen, when the transgene is incorporated in a highly methylated region of the genome, or when more than one copy of the transgene is integrated (Vaucheret et al. 1998). It can also be explained by a transgene inactivation mechanism, in which the plant inactivates the transgene if recognized as a foreign DNA (Finnegan and McElroy 1994), which could happen before the edit occurred and as a result the plants without the edit and with no transgene expression are obtained.

The total amount of anthocyanins in the reddish orange plants obtained from line B2.1 was significantly lower than that in the WT. This is in agreement with the lower anthocyanin content of reddish orange compared with dark red cultivars (Nitarska et al. 2018). The level of pelargonidin remained almost unchanged in the transgenic plants compared with the wild type, only in line B2 was it significantly higher. Simultaneously, line B2.1 has a significantly lower cyanidin level, which indicates reduced F3’H activity compared with the WT (Fig. 6). The presence of cyanidin type anthocyanidins in line B2, however, suggests that the plant is heterozygous and that an allele with unmutated *F3*′*H* is also expressed. The lower anthocyanin content of the reddish orange line and the unchanged pelargonidin content can be explained by the low substrate specificity of the dihydroflavonol 4-reductase of poinsettia for DHK (Nitarska et al. 2018), which creates a bottleneck resulting in a slower anthocyanin formation and, therefore, in lower total anthocyanin concentrations, but because of the reduced F3′H activity an increased ratio of pelargonidin:cyanidin.

As reported for other plants (Jang et al. 2016), the expression of poinsettia *F3*′*H* was not affected by the CRISPR/Cas9 system. The expression level of *Cas9* was much higher in the plants obtained from line B2 compared with plants obtained from lines B158 and B284, which correlates with the mutation efficiency. A positive correlation between *Cas9* expression and mutation frequency was also reported in rice (*Oryza sativa*) (Mikami et al. 2015; Jang et al. 2016).

Cloning of *F3’H* from transgenic plants, expression in yeast and enzyme assay revealed that in line B2 at least three versions of *F3*′*H* are expressed. Version 1, with a sequence identical to the WT, showed no change in protein activity, which explains the presence of cyanidin in the bracts of line B2. Version 2, with insertion of T in position 171, which causes a shift in open reading frame and premature protein termination. The encoded protein was not active, which also supports the hypothesis that the mutation in the *F3*′*H* gene is a cause of change in poinsettia bract colour from red to orange (Nitarska et al. 2018). There is also a third version of *F3*′*H*, where 42 amino acids from the N-terminal of the protein were deleted. The obtained protein is almost not active, with only very slight activity with naringenin being observed. This version of *F3*′*H* was not detected during our NGS studies, probably due to big difference in the size, leading to this fragment being lost during the size selection process.

The CRISPR/Cas9 system is without doubt an interesting alternative for targeted mutagenesis in poinsettia. It should, however, be also taken into account that poinsettia transformation and regeneration is a challenging process and protocol optimization for each variety seems to be essential. Future work will concentrate on self-pollination of line B2 in order to obtain homozygous plants with more intense orange colour, which will accumulate prevalently pelargonidin. We are also planning to try to increase mutation efficiency in lines B158 and B284 by heat stress treatment as was performed by (LeBlanc et al. 2018).

## Conclusion

In this study we reported for the first time successful targeted mutagenesis with the CRISPR/Cas9 system in the commercially important poinsettias. Poinsettias are propagated vegetatively, and selected varieties are frequently marketed as a series with a range of colours. This study puts forward CRISPR/Cas9 as an alternative to the process of irradiated mutation for breeding of colour variation, which is currently the standard practice in commercial poinsettia breeding. We have shown by way of F3′H as an example, that genome editing in poinsettia allows well directed knock-out of specific genes. With increasing knowledge on poinsettia metabolism, targeted knock-in or alteration of specific genes is only a question of time and would be strongly facilitated by the availability of a poinsettia genome. This includes another promising method in the repertoire of ornamental breeding and could be of relevance for other cultures where radiation breeding is frequently used, e.g. chrysanthemum, begonia or dahlia (Anne and Lim 2020). Genome editing methods will become fully exploitable once their status under EU law becomes uncoupled from that of heretofore conventional genetic engineering methods. In case that the EU regulation of genome edited plants will persist over the next decade, the knowledge obtained in this study could also be used to created new colour variants by conventional mutation techniques as e.g. Tilling. To the flavonoid community, this demonstrated that pelargonidin based flower colour can be achieved in an appropriate biochemical background even if DFR shows a low specificity for dihydrokaempferol, as previously hypothesized on the occasion of the escaped orange genetically modified petunia.

## Supplementary Information

Below is the link to the electronic supplementary material.

Supplementary file1 (DOCX 22 kb)

## Data Availability

All data generated or analyzed during this study are included in this published article and its supplementary information file. Primary datasets are available from the corresponding author on reasonable request.
