# Supplementary material for: First genome edited poinsettias: targeted mutagenesis of flavonoid 3′-hydroxylase using CRISPR/Cas9 results in a colour shift
Source: Plant Cell Tissue Organ Cult. 2021 May 26;147(1):49–60. doi: 10.1007/s11240-021-02103-5 (PMC8550517; doi:10.1007/s11240-021-02103-5)
Supplement: Supplementary file 1 — Supplementary file1 (DOCX 22 kb) [file 11240_2021_2103_MOESM1_ESM.docx]

| **Name** | **Sequence 5’ – 3’** | **Product size, purpose** |
| --- | --- | --- |
| nptII-F | ACAAGATGGATTGCACGCAGG | 780 bp, transgene detection |
| nptII-R | AACTCGTCAAGAAGGCGATAG |  |
| Cas9-F1 | ATGAAGAGGAACTACATCCTCGG | 564 bp, transgene detection |
| Cas9-F2 | CTTCTGCACCTTGAGGAGCT |  |
| Cas9-F2 | GGATAATGGACCTGTGATCAAGAAG | 577 bp, transgene detection |
| Cas9-R2 | CTTCCTCTTCTTCTTAGGATCAGC |  |
| gRNAfull-F | ACGCGTCCTGAGGCTTTTTTTC | 510 bp, transgene detection |
| gRNAfull-R | CAGGACGCGTAAAAATCTCGC |  |
| EpActin | GCTCAGTCCAAGAGAGGTATTT | 243 bp, transgene detection |
| EpActin | AGCCTGAATAGCGACATACATAG |  |
| EpGAPDH-F | GAGACGATGTGGAGCTTGTT | 766 bp, transgene detection |
| EpGAPDH-R | TTTCCCTCAGATTCTGCCTTTAT |  |
| EpF3’HpYes-F | ATGTTACCACTCTTTGCGTTTACC | 265 bp, sequence analysis |
| EpF3’Hcrispr_R | CTTTCAAGAACTGGGCAGCAAC |  |
| qEpActin2-F | CTGTTCCAGCCATCTCTCATT | 127 bp, qPCR efficiency 2.06 |
| qEpActin2-R | AACCGCCACTCAGAACTATG |  |
| qEpEF1A-F | AAGATGATTCCCACCAAGCCCA | 72 bp, qPCR efficiency 2.01 |
| qEpEF1A-R | CACAGCAAAACGACCCAGAGGA |  |
| qEpF3’H2-F | AATCAACGGCTACCACATCC | 91 bp, qPCR efficiency 2.02 |
| qepF3’H2-R | CAGCGGCTCTTTCCATACTT |  |
| qCas9-F | CAACGAGGGAAGAAGGTCTAAG | 110 bp, qPCR efficiency 2.04 |
| qCas9-R | GATCGGTGAGGAGGTTGTAATC |  |
| EpF3’HpYes-F | ATGTTACCACTCTTTGCGTTTACC | 1533 bp, F3’H cloning into pYES  vector |
| EpF3’HpYes-R | TCAAACAGGAGCAGCATAGACTA |  |

Suppl. Table S1. Primers used during the study

WT-K4 ATGTTACCACTCTTTGCGTTTACCATTTTTTCTGCCATTTTCATTTCCTTTTT---CTTC 57 2-K2-v1 ATGTTACCACTCTTTGCGTTTACCATTTTTTCTGCCATTTTCATTTCCTTTTT---CTTC 57 2-K1-v2 ATGTTACCACTCTTTGCGTTTACCATTTTTTCTGCCATTTTCATTTCCTTTTT---CTTC 57 2-K3-v3 ATGTTACCACTCTTTGCGTTTACCATTTTTTCTGCCATTTTCATTTCCTTTTTTTTCTTC 60 158-K6 ATGTTACCACTCTTTGCGTTTACCATTTTTTCTGCCATTTTCATTTCCTTTTT---CTTC 57 284-K3 ATGTTACCACTCTTTGCGTTTACCATTTTTTCTGCCATTTTCATTTCCTTTTTTTTCTTC 60

WT-K4 TTCTTTTTTCGCCGTACCTCTCGCCCCCCTCTTCCTCCCGGTCCTAGACCACTGCCTGTA 117 2-K2-v1 TTCTTTTTTCGCCGTACCTCTCGCCCCCCTCTTCCTCCCGGTCCTAGACCACTGCCTGTA 117 2-K1-v2 TTCTTTTTTCGCCGTACCTCTCGCCCCCCTCTTCCTCCCGGTCCTAGACCACTGCCTGTA 117 2-K3-v3 TTCTTTTTTCGCCGTACCTCTCGCCCCCCTCTTCCTCCCGGTCCTAGACCAC-------- 90 158-K6 TTCTTTTTTCGCCGTACCTCTCGCCCCCCTCTTCCTCCCGGTCCTAGACCACTGCCTGTA 117 284-K3 TTCTTTTTTCGCCGTACCTCTCGCCCCCCTCTTCCTCCCGGTCCTAGACCACTGCCTGTA 120

WT-K4 ATTGGAAACCTGCCTCATTTAGGCCCCAAACCCCACCAGTCAATAGCCTCCT-TGGCTCG 176 2-K2-v1 ATTGGAAACCTGCCTCATTTAGGCCCCAAACCCCACCAGTCAATAGCCTCCT-TGGCTCG 176 2-K1-v2 ATTGGAAACCTGCCTCATTTAGGCCCCAAACCCCACCAGTCAATAGCCTCCTTTGGCTCG 177 2-K3-v3 ------------------------------------------------------------ 90 158-K6 ATTGGAAACCTGCCTCATTTAGGCCCCAAACCCCACCAGTCAATAGCCTCCT-TGGCTCG 176 284-K3 ATTGGAAACCTGCCTCATTTAGGCCCCAAACCCCACCAGTCAATAGCCTCCT-TGGCTCG 179

WT-K4 GGTTTATGGCCCCCTTATGCACCTCCGTATGGGCTTTGTCGACGTCGTTGTGGCGGCGTC 236 2-K2-v1 GGTTTATGGCCCCCTTATGCACCTCCGTATGGGCTTTGTCGACGTCGTTGTGGCGGCGTC 236 2-K1-v2 GGTTTATGGCCCCCTTATGCACCTCCGTATGGGCTTTGTCGACGTCGTTGTGGCGGCGTC 237 2-K3-v3 -----------------------------------------------------------C 113 158-K6 GGTTTATGGCCCCCTTATGCACCTCCGTATGGGCTTTGTCGACGTCGTTGTGGCGGCGTC 236 284-K3 GGTTTATGGCCCCCTTATGCACCTCCGTATAGGCTTTGTCGACGTCGTTGTGGCGGCGTC 239

WT-K4 GGCGTCCGTTGCTGCCCAGTTCTTGAAAGCTCATGACGCTAATTTCTCGAGCCGGCCGCC 296 2-K2-v1 GGCGTCCGTTGCTGCCCAGTTCTTGAAAGCTCATGACGCTAATTTCTCGAGCCGGCCGCC 296 2-K1-v2 GGCGTCCGTTGCTGCCCAGTTCTTGAAAGCTCATGACGCTAATTTCTCGAGCCGGCCGCC 297 2-K3-v3 GGCGTCCGTTGCTGCCCAGTTCTTGAAAGCTCATGACGCTAATTTCTCGAGCCGGCCGCC 173 158-K6 GGCGTCCGTTGCTGCCCAGTTCTTGAAAGCTCATGACGCTAATTTCTCGAGCCGGCCGCC 296 284-K3 GGCGTCCGTTGCTGCCCAGTTCTTGAAAGCTCATGACGCTAATTTCTCGAGCCGGCCGCC 299

WT-K4 TAATTCGGGTGCTAAGTATGTTGCTTATAATTACCAAGATCTTGTTTTTGCCCCGTACGG 356 2-K2-v1 TAATTCGGGTGCTAAGTATGTTGCTTATAATTACCAAGATCTTGTTTTTGCCCCGTACGG 356 2-K1-v2 TAATTCGGGTGCTAAGTATGTTGCTTATAATTACCAAGATCTTGTTTTTGCCCCGTACGG 357 2-K3-v3 TAATTCGGGTGCTAAGTATGTTGCTTATAATTACCAAGATCTTGTTTTTGCCCCGTACGG 233 158-K6 TAATTCGGGTGCTAAGTATGTTGCTTATAATTACCAAGATCTTGTTTTTGCCCCGTACGG 356 284-K3 TAATTCGGGTGCTAAGTATGTTGCTTATAATTACCAAGATCTTGTTTTTGCCCCGTACGG 359

WT-K4 ACCTCGCTGGCGCATGCTCAGGAAAATCAGTGCCGTGCATCTCTTCTCGGCTAAGGCCTT 416 2-K2-v1 ACCTCGCTGGCGCATGCTCAGGAAAATCAGTGCCGTGCATCTCTTCTCGGCTAAGGCCTT 416 2-K1-v2 ACCTCGCTGGCGCATGCTCAGGAAAATCAGTGCCGTGCATCTCTTCTCGGCTAAGGCCTT 417 2-K3-v3 ACCTCGCTGGCGCATGCTCAGGAAAATCAGTGCCGTGCATCTCTTCTCGGCTAAGGCCTT 293 158-K6 ACCTCGCTGGCGCATGCTCAGGAAAATCAGTGCCGTGCATCTCTTCTCGGCTAAGGCCTT 416 284-K3 ACCTCGCTGGCGCATGCTCAGGAAAATCAGTGCCGTGCATCTCTTCTCGGCTAAGGCCTT 419

WT-K4 GGATGATTTCCGCCATGTTAGACAGGAAGAAGTGGCAATCCTTGTACGGTCTCTAGTAAG 476 2-K2-v1 GGATGATTTCCGCCATGTTAGACAGGAAGAAGTGGCAATCCTTGTACGGTCTCTAGTAAG 476 2-K1-v2 GGATGATTTCCGCCATGTTAGACAGGAAGAAGTGGCAATCCTTGTACGGTCTCTAGTAAG 477 2-K3-v3 GGATGATTTCCGCCATGTTAGACAGGAAGAAGTGGCAATCCTTGTACGGTCTCTAGTAAG 353 158-K6 GGATGATTTCCGCCATGTTAGACAGGAAGAAGTGGCAATCCTTGTACGGTCTCTAGTAAG 476 284-K3 GGATGATTTCCGCCATGTTAGACAGGAAGAAGTGGCAATCCTTGTACGGTCTCTAGTAAG 479

WT-K4 TTCCGGGCACGAAAGAGCGGTCAATTTAGGGCATCTGGTGAACCTGTGCGCCACAAATGC 536 2-K2-v1 TTCCGGGCACGAAAGAGCGGTCAATTTAGGGCATCTGGTGAACCTGTGCGCCACAAATGC 536 2-K1-v2 TTCCGGGCACGAAAGAGCGGTCAATTTAGGGCATCTGGTGAACCTGTGCGCCACAAATGC 537 2-K3-v3 TTCCGGGCACGAAAGAGCGGTCAATTTAGGGCATCTGGTGAACTTGTGCGCCACAAATGC 413 158-K6 TTCCGGGCACGAAAGAGCGGTCAATTTAGGGCATCTGGTGAACCTGTGCGCCACAAATGC 536 284-K3 TTCCGGGCACGAAAGAGCGGTCAATTTAGGGCATCTGGTGAACCTGTGCGCCACAAATGC 539

WT-K4 ACTGGCACGCGTAATGATTGGCAGAAGAGTATTCAGCGACAGCGGTGATCCGAAGGCCGA 596 2-K2-v1 ACTGGCACGCGTAATGATTGGCAGAAGAGTATTCAGCGACAGCGGTGATCCGAAGGCCGA 596 2-K1-v2 ACTGGCACGCGTAATGATTGGCAGAAGAGTATTCAGCGACAGCGGTGATCCGAAGGCCGA 597 2-K3-v3 ACTGGCACGCGTAATGATTGGCAGAAGAGTATTCAGCGACAGCGGTGATCCGAAGGCCGA 473 158-K6 ACTGGCACGCGTAATGATTGGCAGAAGAGTATTCAGCGACAGCGGTGATCCGAAGGCCGA 596 284-K3 ACTGGCACGCGTAATGATTGGCAGAAGAGTATTCAGCGACAGCGGTGATCCGAAGGCCGA 599

WT-K4 CGAGTTCAAGTCAATGGTGGTGGAACTGATGAGACTTGCCGGAGTATTCAATATAGGGGA 656 2-K2-v1 CGAGTTCAAGTCAATGGTGGTGGAACTGATGAGACTTGCCGGAGTATTCAATATAGGGGA 656 2-K1-v2 CGAGTTCAAGTCAATGGTGGTGGAACTGATGAGACTTGCCGGAGTATTCAATATAGGGGA 657 2-K3-v3 CGAGTTCAAGTCAATGGTGGTGGAACTGATGAGACTTGCCGGAGTATTCAATATAGGGGA 533 158-K6 CGAGTTCAAGTCAATGGTGGTGGAACTGATGAGACTTGCCGGAGTATTCAATATAGGGGA 656 284-K3 CGAGTTCAAGTCAATGGTGGTGGAACTGATGAGACTTGCCGGAGTATTCAATATAGGGGA 659

WT-K4 TTTTATTCCGGCACTGGAGTGGCTGGATTTACAGCGAGTAGCAGCTAAAATGAAGAAACT 716 2-K2-v1 TTTTATTCCGGCACTGGAGTGGCTGGATTTACAGCGAGTAGCAGCTAAAATGAAGAAACT 716 2-K1-v2 TTTTATTCCGGCACTGGAGTGGCTGGATTTACAGCGAGTAGCAGCTAAAATGAAGAAACT 717 2-K3-v3 TTTTATTCCGGCACTGGAGTGGCTGGATTTACAGCGAGTAGCAGCTAAAATGAAGAAACT 593 158-K6 TTTTATTCCGGCACTGGAGTGGCTGGATTTACAGCGAGTAGCAGCTAAAATGAAGAAACT 716 284-K3 TTTTATTCCGGCACTGGAGTGGCTGGATTTACAGCGAGTAGCAGCTAAGATGAAGAAACT 719

WT-K4 CCATAAGAGATTCGATGCGTTTTTGACTGAAATCGTCGAGGAACACAAGAGTAACAAAGG 776 2-K2-v1 CCATAAGAGATTCGATGCGTTTTTGACTGAAATCGTCGAGGAACACAAGAGTAACAAAGG 776 2-K1-v2 CCATAAGAGATTCGATGCGTTTTTGACTGAAATCGTCGAGGAACACAAGAGTAACAAAGG 777 2-K3-v3 CCATAAGAGATTCGATGCGTTTTTGACTGAAATCGTCGAGGAACACAAGAGTAACAAAGG 653 158-K6 CCATAAGAGATTCGATGCGTTTTTGACTGAAATCGTCGAGGAACACAAGAGTAACAAAGG 776 284-K3 CCATAAGAGATTCGATGCGTTTTTGACTGAAATCGTCGAGGAACACAAGAGTAACAGAGG 779

WT-K4 AGAGTCAACTCACAGAGACATGTTGACTACTTTAATCTCGTTAAAGGAGGAAGAAGCTGA 836 2-K2-v1 AGAGTCAACTCACAGAGACATGTTGACTACTTTAATCTCGTTAAAGGAGGAAGAAGCTGA 836 2-K1-v2 AGAGTCAACTCACAGAGACATGTTGACTACTTTAATCTCGTTAAAGGAGGAAGAAGCTGA 837 2-K3-v3 AGAGTCAACTCACAGAGACATGTTGACTACTTTAATCTCGTTAAAGGAGGAAGAAGCTGA 713 158-K6 AGAGTCAACTCACAGAGACATGTTGACTACTTTAATCTCGTTAAAGGAGGAAGAAGCTGA 836 284-K3 AGAGTCAACTCACAGAGACATGTTGACTACTTTAATCTCGTTAAAGGAGGAAGAAGCTGA 839

WT-K4 TGACGGTGAGGGAGGGAAAATCACTGACACCGAAATTAAAGCCCTGCTTCTGAACATGTT 896 2-K2-v1 TGACGGTGAGGGAGGGAAAATCACTGACACCGAAATTAAAGCCCTGCTTCTGAACATGTT 896 2-K1-v2 TGACGGTGAGGGAGGGAAAATCACTGACACCGAAATTAAAGCCCTGCTTCTGAACATGTT 897 2-K3-v3 TGACGGTGAGGGAGGGAAAATCACTGACACCGAAATTAAAGCCCTGCTTCTGAACATGTT 773 158-K6 TGACGGTGAGGGAGGGAAAATCACTGACACCGAAATTAAAGCCCTGCTTCTGAACATGTT 896 284-K3 TGACGGTGAGGGAGGGAAAATCACTGACACCGAAATTAAAGCCCTGCTTCTGAACATGTT 899

WT-K4 TGCAGCAGGCACCGACACTACATCAAGCACGGTTGAGTGGGCCATTGCTGAGCTCATCAG 956 2-K2-v1 TGCAGCAGGCACCGACACTACATCCAGCACAGTTGAGTGGGCCATTGCTGAGCTCATCAG 956 2-K1-v2 TGCAGCAGGCACCGACACTACATCCAGCACAGTTGAGTGGGCCATTGCTGAGCTCATCAG 957 2-K3-v3 TGCAGCAGGCACCGACACTACATCAAGCACGGTTGAGTGGGCCATTGCTGAGCTCATCAG 833 158-K6 TGCAGCAGGCACCGACACTACATCAAGCACGGTTGAGTGGGCCATTGCTGAGCTCATCAG 956 284-K3 TGCAGCAGGCACCGACACTACATCAAGCACGGTTGAGTGGGCCATTGCTGAGCTCATCAG 959

WT-K4 GCACCCCAAAATACTAACCAAACTCCGGCGAGAACTCGACTCCGTCGTCGGCGCCGATTG 1016 2-K2-v1 GCACCCCAAAATACTAACCAAACTCCGGCGAGAACTCGACTCCGTCGTCGGCGCCGATTG 1016 2-K1-v2 GCACCCCAAAATACTAACCAAACTCCGGCAAGAACTCGACTCCGTCGTCGGCGCCGATTG 1017 2-K3-v3 GCACCCCAAAATACTAACCAAACTCCGGCAAGAACTCGACTCCGTCGTCGGCGCCGATTG 893 158-K6 GCACCCCAAAATACTAACCAAACTCCGGCAAGAACTCGACTCCGTCGTCGGCGCCGATTG 1016 284-K3 GCACCCCAAAATACTAACCAAACTCCGGCAAGAACTCGACTCCGTCGTCGGCGCCGATTG 1019

WT-K4 TCTCGTAACCGAGCTAGACATCACTCAACTCCCCTACCTCCAAGCCGTCGTCAAAGAAAC 1076 2-K2-v1 TCTCGTAACCGAGCTAGACATCACTCAACTCCCCTACCTCCAAGCCGTCGTCAAAGAAAC 1076 2-K1-v2 TCTCGTAACCGAGCTAGACATCACTCAACTCCCCTACCTCCAAGCCGTCGTCAAAGAAAC 1077 2-K3-v3 TCTCGTAACCGAGCTAGACATCACTCAACTCCCCTACCTCCAAGCCGTCGTCAAAGAAAC 953 158-K6 TCTCGTAACCGAGCTAGACATCACTCAACTCCCCTACCTCCAAGCCGTCGTCAAAGAAAC 1076 284-K3 TCTCGTAACCGAGCTAGACATCACTCAACTCCCCTACCTCCAAGCCGTCGTCAAAGAAAC 1079

WT-K4 CTTCCGCCTCCACCCATCAACTCCCCTCTCTCTCCCTCGAATGGCGGCCGAAAGCTGCGA 1136 2-K2-v1 CTTCCGCCTCCACCCATCAACTCCCCTCTCTCTCCCTCGAATGGCGGCCGAAAGCTGCGA 1136 2-K1-v2 CTTCCGCCTCCACCCATCAACTCCCCTCTCTCTCCCTCGAATGGCGGCCGAAAGCTGCGA 1137 2-K3-v3 CTTCCGCCTCCACCCATCAACTCCCCTCTCTCTCCCTCGAATGGCGGCCGAAAGCTGCGA 1013 158-K6 CTTCCGCCTCCACCCATCAACTCCCCTCTCTCTCCCTCGAATGGCGGCCGAAAGCTGCGA 1136 284-K3 CTTCCGCCTCCACCCATCAACTCCCCTCTCTCTCCCTCGAATGGCGGCCGAAAGCTGCGA 1139

WT-K4 AATCAACGGCTACCACATCCCAAAAGGCGCCACGCTTCTGGTCAACGTGTGGGCAATAGC 1196 2-K2-v1 AATCAACGGCTACCACATCCCAAAAGGCGCCACGCTTCTGGTCAACGTGTGGGCAATAGC 1196 2-K1-v2 AATCAACGGCTACCACATCCCAAAAGGCGCCACGCTTCTGGTCAACGTGTGGGCAATAGC 1197 2-K3-v3 AATCAACGGCTACCACATCCCAAAAGGCGCCACGCTTCTGGTCAACGTGTGGGCAATAGC 1073 158-K6 AATCAACGGCTACCACATCCCAAAAGGCGCCACGCTTCTGGTCAACGTGTGGGCAATAGC 1196 284-K3 AATCAACGGCTACCACATCCCAAAAGGCGCCACGCTTCTGGTCAACGTGTGGGCAATAGC 1199

WT-K4 TCGCGATCCAGAAGTATGGAAAGAGCCGCTGGAGTTTCGACCGGAGAGGTTTCTCGCCGG 1256 2-K2-v1 TCGCGATCCAGAAGTATGGAAAGAGCCGCTGGAGTTTCGACCGGAGAGGTTTCTCGCCGG 1256 2-K1-v2 TCGCGATCCAGAAGTATGGAAAGAGCCGCTGGAGTTTCGACCGGAGAGGTTTCTCGCCGG 1257 2-K3-v3 TCGCGATCCAGAAGTATGGAAAGAGCCGCTGGAGTTTCGACCGGAGAGGTTTCTCGCCGG 1133 158-K6 TCGCGATCCAGAAGTATGGAAAGAGCCGCTGGAGTTTCGACCGGAGAGGTTTCTCGCCGG 1256 284-K3 TCGCGATCCAGAAGTATGGAAAGAGCCGCTGGAGTTTCGACCGGAGAGGTTTCTCGCCGG 1259

WT-K4 CGGAGAAAGGCCGAACGCCGACGTGAAAGGGACGGATTTTGAGGTGATTCCGTTTGGGGC 1316 2-K2-v1 CGGAGAAAGGCCGAACGCCGACGTGAAAGGGACGGATTTTGAGGTGATTCCGTTTGGGGC 1316 2-K1-v2 CGGAGAAAGGCCGAACGCCGACGTGAAAGGGACGGATTTTGAGGTGATTCCGTTTGGGGC 1317 2-K3-v3 CGGAGAAAGGCCGAACGCCGACGTGAAAGGGACGGATTTTGAGGTGATTCCGTTTGGGGC 1193 158-K6 CGGAGAAAGGCCGAACGCCGACGTGAAAGGGACGGATTTTGAGGTGATTCCGTTTGGGGC 1316 284-K3 CGGAGAAAGGCCGAACGCCGACGTGAAAGGGACGGATTTTGAGGTGATTCCGTTTGGGGC 1319

WT-K4 AGGGCGGAGAATTTGCGCAGGGATGAATTTAGGGTTAGTAATGGTTCATCTGCTTATTGC 1376 2-K2-v1 AGGGCGGAGAATTTGCGCAGGGATGAATTTAGGGTTAGTAATGGTTCATCTGCTTATTGC 1376 2-K1-v2 AGGGCGGAGAATTTGCGCAGGGATAAATTTAGGGTTAGTAATGGTTCATCTGCTTATTGC 1377 2-K3-v3 AGGGCGGAGAATTTGCGCAGGGATGAATTTAGGGTTAGTAATGGTTCATCTGCTTATTGC 1253 158-K6 AGGGCGGAGAATTTGCGCAGGGATGAATTTAGGGTTAGTAATGGTTCATCTGCTTATTGC 1376 284-K3 AGGGCGGAGAATTTGCGCAGGGATGAATTTAGGGTTAGTAATGGTTCATCTGCTTATTGC 1379

WT-K4 GAGTTTAGTACAAGGATTTGAATGGGAATTGGAAAGGGAAAAACCAGAGAAATTGAACAT 1436 2-K2-v1 GAGTTTAGTACAAGGATTTGAATGGGAATTGGAAAGGGAAAAACCAGAGAAATTGAACAT 1436 2-K1-v2 GAGTTTAGTACAAGGATTTGAATGGGAATTGGAAAGGGAAAAACCAGAGAAATTGAACAT 1437 2-K3-v3 GAGTTTAGTACAAGGATTTGAATGGGAATTGGAAAGGGAAAAACCAGAGAAATTGAACAT 1313 158-K6 GAGTTTAGTACAAGGATTTGAATGGGAATTGGAAAGGGAAAAACCAGAGAAATTGAACAT 1436 284-K3 GAGTTTAGTACAAGGATTTGAATGGGAATTGGAAAGGGAAAAACCAGAGAAATTGAACAT 1439

WT-K4 GGAGGAAGCTTATGGGCTGACCTTACAACGACTTGAGCCATTGATGGTGTACCCAAACCC 1496 2-K2-v1 GGAGGAAGCTTATGGGCTGACCTTACAACGACTTGAGCCATTGATGGTGTACCCAAACCC 1496 2-K1-v2 GGAGGAAGCTTATGGGCTGACCTTACAACGACTTGAGCCATTGATGGTGTACCCAAACCC 1497 2-K3-v3 GGAGGAAGCTTATGGGCTGACCTTACAACGACTTGAGCCATTGATGGTGTACCCAAACCC 1373 158-K6 GGAGGAAGCTTATGGGCTGACCTTACAACGACTTGAGCCATTGATGGTGTACCCAAACCC 1496 284-K3 GGAGGAAGCTTATGGGCTGACCTTACAACGACTTGAGCCATTGATGGTGTACCCAAACCC 1499

WT-K4 TAGGTTGTCGTCTCTAGTCTATGCTGCTCCTGTTTGA 1533

2-K2-v1 TAGGTTGTCGTCTCTAGTCTATGCTGCTCCTGTTTGA 1533

2-K1-v2 TAGGTTGTCGTCTCTAGTCTATGCTGCTCCTGTTTGA 1534

2-K3-v3 TAGGTTGTCGTCTCTAGTCTATGCTGCTCCTGTTTGA 1410

158-K6 TAGGTTGTCGTCTCTAGTCTATGCTGCTCCTGTTTGA 1533

284-K3 TAGGTTGTCGTCTCTAGTCTATGCTGCTCCTGTTTGA 1536

Suppl. Fig. S1 Multiple nucleotide sequence alignment of *F3’H*s from transgenic poinsettia and WT. Black highlight marks insertion of T in position 170 in 2-K1-v2

WT-K4 MLPLFAFTIFSAIFISFFF-FFFRRTSRPPLPPGPRPLPVIGNLPHLGPKPHQSIASLAR 59 2-K2-v1 MLPLFAFTIFSAIFISFFF-FFFRRTSRPPLPPGPRPLPVIGNLPHLGPKPHQSIASLAR 59 2-K1-v2 MLPLFAFTIFSAIFISFFF-FFFRRTSRPPLPPGPRPLPVIGNLPHLGPKPHQSIASFGS 59 2-K3-v3 MLPLFAFTIFSAIFISFFFFFFFRRTSRPPLPPGPRPP---------------------- 38 158-K6 MLPLFAFTIFSAIFISFFF-FFFRRTSRPPLPPGPRPLPVIGNLPHLGPKPHQSIASLAR 59 284-K3 MLPLFAFTIFSAIFISFFFFFFFRRTSRPPLPPGPRPLPVIGNLPHLGPKPHQSIASLAR 60

WT-K4 VYGPLMHLRMGFVDVVVAASASVAAQFLKAHDANFSSRPPNSGAKYVAYNYQDLVFAPYG 119 2-K2-v1 VYGPLMHLRMGFVDVVVAASASVAAQFLKAHDANFSSRPPNSGAKYVAYNYQDLVFAPYG 119 2-K1-v2 GLWPPYAPPYGLCRRRCGGVGVRCCPVLESS----------------------------- 90 2-K3-v3 --------------------ASVAAQFLKAHDANFSSRPPNSGAKYVAYNYQDLVFAPYG 78 158-K6 VYGPLMHLRMGFVDVVVAASASVAAQFLKAHDANFSSRPPNSGAKYVAYNYQDLVFAPYG 119 284-K3 VYGPLMHLRIGFVDVVVAASASVAAQFLKAHDANFSSRPPNSGAKYVAYNYQDLVFAPYG 120

WT-K4 PRWRMLRKISAVHLFSAKALDDFRHVRQEEVAILVRSLVSSGHERAVNLGHLVNLCATNA 179 2-K2-v1 PRWRMLRKISAVHLFSAKALDDFRHVRQEEVAILVRSLVSSGHERAVNLGHLVNLCATNA 179 2-K1-v2 ------------------------------------------------------------ 90

2-K3-v3 PRWRMLRKISAVHLFSAKALDDFRHVRQEEVAILVRSLVSSGHERAVNLGHLVNLCATNA 138 158-K6 PRWRMLRKISAVHLFSAKALDDFRHVRQEEVAILVRSLVSSGHERAVNLGHLVNLCATNA 179 284-K3 PRWRMLRKISAVHLFSAKALDDFRHVRQEEVAILVRSLVSSGHERAVNLGHLVNLCATNA 180

WT-K4 LARVMIGRRVFSDSGDPKADEFKSMVVELMRLAGVFNIGDFIPALEWLDLQRVAAKMKKL 239 2-K2-v1 LARVMIGRRVFSDSGDPKADEFKSMVVELMRLAGVFNIGDFIPALEWLDLQRVAAKMKKL 239 2-K1-v2 ------------------------------------------------------------ 90

2-K3-v3 LARVMIGRRVFSDSGDPKADEFKSMVVELMRLAGVFNIGDFIPALEWLDLQRVAAKMKKL 198 158-K6 LARVMIGRRVFSDSGDPKADEFKSMVVELMRLAGVFNIGDFIPALEWLDLQRVAAKMKKL 239 284-K3 LARVMIGRRVFSDSGDPKADEFKSMVVELMRLAGVFNIGDFIPALEWLDLQRVAAKMKKL 240

WT-K4 HKRFDAFLTEIVEEHKSNKGESTHRDMLTTLISLKEEEADDGEGGKITDTEIKALLLNMF 299 2-K2-v1 HKRFDAFLTEIVEEHKSNKGESTHRDMLTTLISLKEEEADDGEGGKITDTEIKALLLNMF 299 2-K1-v2 ------------------------------------------------------------ 90

2-K3-v3 HKRFDAFLTEIVEEHKSNKGESTHRDMLTTLISLKEEEADDGEGGKITDTEIKALLLNMF 258 158-K6 HKRFDAFLTEIVEEHKSNKGESTHRDMLTTLISLKEEEADDGEGGKITDTEIKALLLNMF 299 284-K3 HKRFDAFLTEIVEEHKSNRGESTHRDMLTTLISLKEEEADDGEGGKITDTEIKALLLNMF 300

WT-K4 AAGTDTTSSTVEWAIAELIRHPKILTKLRRELDSVVGADCLVTELDITQLPYLQAVVKET 359 2-K2-v1 AAGTDTTSSTVEWAIAELIRHPKILTKLRRELDSVVGADCLVTELDITQLPYLQAVVKET 359 2-K1-v2 ------------------------------------------------------------ 90

2-K3-v3 AAGTDTTSSTVEWAIAELIRHPKILTKLRQELDSVVGADCLVTELDITQLPYLQAVVKET 318 158-K6 AAGTDTTSSTVEWAIAELIRHPKILTKLRQELDSVVGADCLVTELDITQLPYLQAVVKET 359 284-K3 AAGTDTTSSTVEWAIAELIRHPKILTKLRQELDSVVGADCLVTELDITQLPYLQAVVKET 360

WT-K4 FRLHPSTPLSLPRMAAESCEINGYHIPKGATLLVNVWAIARDPEVWKEPLEFRPERFLAG 419 2-K2-v1 FRLHPSTPLSLPRMAAESCEINGYHIPKGATLLVNVWAIARDPEVWKEPLEFRPERFLAG 419 2-K1-v2 ------------------------------------------------------------ 90

2-K3-v3 FRLHPSTPLSLPRMAAESCEINGYHIPKGATLLVNVWAIARDPEVWKEPLEFRPERFLAG 378 158-K6 FRLHPSTPLSLPRMAAESCEINGYHIPKGATLLVNVWAIARDPEVWKEPLEFRPERFLAG 419 284-K3 FRLHPSTPLSLPRMAAESCEINGYHIPKGATLLVNVWAIARDPEVWKEPLEFRPERFLAG 420

WT-K4 GERPNADVKGTDFEVIPFGAGRRICAGMNLGLVMVHLLIASLVQGFEWELEREKPEKLNM 479 2-K2-v1 GERPNADVKGTDFEVIPFGAGRRICAGMNLGLVMVHLLIASLVQGFEWELEREKPEKLNM 479 2-K1-v2 ------------------------------------------------------------ 90

2-K3-v3 GERPNADVKGTDFEVIPFGAGRRICAGMNLGLVMVHLLIASLVQGFEWELEREKPEKLNM 438 158-K6 GERPNADVKGTDFEVIPFGAGRRICAGMNLGLVMVHLLIASLVQGFEWELEREKPEKLNM 479 284-K3 GERPNADVKGTDFEVIPFGAGRRICAGMNLGLVMVHLLIASLVQGFEWELEREKPEKLNM 480

WT-K4 EEAYGLTLQRLEPLMVYPNPRLSSLVYAAPV 510

2-K2-v1 EEAYGLTLQRLEPLMVYPNPRLSSLVYAAPV 510

2-K1-v2 ------------------------------- 90

2-K3-v3 EEAYGLTLQRLEPLMVYPNPRLSSLVYAAPV 469

158-K6 EEAYGLTLQRLEPLMVYPNPRLSSLVYAAPV 510

284-K3 EEAYGLTLQRLEPLMVYPNPRLSSLVYAAPV 511

Suppl. Fig. S2 Multiple amino acid sequences alignment of F3’H from transgenic poinsettia and WT
